# Newly proposed insulin resistance indexes called TyG-NC and TyG-NHtR show efficacy in diagnosing the metabolic syndrome

**DOI:** 10.1007/s40618-021-01608-2

**Published:** 2021-06-16

**Authors:** M. Mirr, D. Skrypnik, P. Bogdański, M. Owecki

**Affiliations:** 1 Department of Public Health, Poznan University of Medical Sciences, Rokietnicka St. 4, 60-806 Poznan, Poland; 2 Department of Treatment of Obesity, Metabolic Disorders and Clinical Dietetics, Poznan University of Medical Sciences, Szamarzewskiego St. 82/84, 60-569 Poznan, Poland

**Keywords:** Insulin resistance, Metabolic syndrome, Obesity

## Abstract

**Purpose:**

Obesity and insulin resistance are considered cardinal to the pathophysiology of metabolic syndrome. Several simple indexes of insulin resistance calculated from biochemical or anthropometric variables have been proposed. The study aimed to assess the diagnostic accuracy of indirect insulin resistance indicators in detecting metabolic syndrome in non-diabetic patients, including TG/HDLc, METS-IR, TyG, TyG-BMI, TyG-WC, TyG-WHtR, and new indicators TyG-NC (TyG-neck circumference) and TyG-NHtR (Tyg-neck circumference to height ratio).

**Methods:**

The diagnostic accuracy of eight insulin resistance indexes was assessed using the receiver operating characteristic curves (ROC curves) in 665 adult non-diabetic patients. Then, the analysis was performed after the division into groups with proper body mass index, overweight and obese.

**Results:**

All indexes achieved significant diagnostic accuracy, with the highest AUC (area under the curve) for TyG (0.888) and Tg/HDLc (0.874). The highest diagnostic performance in group with the proper body mass index was shown for TyG (0.909) and TyG-BMI (0.879). The highest accuracy in the group of overweight individuals was presented by TyG (0.884) and TG/HDLc (0.855). TG/HDLc and TyG showed the highest AUC (0.880 and 0.877, respectively) in the group with obesity. Both TyG-NC and TyG-NHtR reached significant areas under the curve, which makes them useful diagnostic tests in metabolic syndrome.

**Conclusions:**

Indirect indices of insulin resistance, including proposed TyG-NC and TyG-NHtR, show an essential diagnostic value in diagnosing metabolic syndrome. TyG and TG/HDLc seem to be the most useful in the Caucasian population.

## Introduction

Metabolic syndrome is a set of features that increase the risk of developing cardiovascular diseases and diabetes [1]. Obesity and insulin resistance are considered cardinal to the pathophysiology of metabolic syndrome [1, 2]. Insulin resistance (IR) is a state of decreased tissue sensitivity to insulin, a glycemic-lowering hormone [3]. Insulin resistance is considered a strong risk factor not only for type 2 diabetes but also for cardiovascular complications, including hypertension and non-alcoholic fatty liver disease (NAFLD) [3–5]. The hyperinsulinemic euglycemic clamp (HEC) is the gold standard in assessing the insulin sensitivity of peripheral tissues [4, 6]. This method consists of continuous insulin infusion until the serum concentration of 100 mIU/L is achieved and maintained, and simultaneous intravenous glucose infusion [4, 7]. During exogenous hyperinsulinemia, insulin production by the pancreas and the hepatic glucose production is blocked, and the amount of glucose administered reflects its tissue uptake, and, thus, indirectly insulin sensitivity [4, 8]. However, because this method is complicated, time- and resource-consuming, insulin resistance is most often assessed using simpler indicators [4, 6].

A commonly used indicator that strongly correlates with insulin resistance assessed by HEC is the homeostatic model assessment for insulin resistance (HOMA-IR), calculated based on fasting glucose and insulin levels [7]. This method’s use is also limited in everyday practice by the relatively high cost of measuring insulin concentration [9]. Several simple indexes of insulin resistance calculated from biochemical or anthropometric variables have been proposed [4, 9]. The triglyceride-glucose (TyG) index derived from circulating triglycerides and glucose concentrations was shown to be comparable or even more predictive than HOMA-IR in assessing the risk of insulin resistance-related conditions [5, 10] TyG strongly correlates with insulin resistance assessed by HEC and shows high sensitivity and specificity in the diagnosis of insulin resistance [11].

The diagnostic value of TyG in the diagnosis of metabolic syndrome has also been shown [12]. In recent years, several indices calculated as products of TyG and anthropometric indices have been proposed, including TyG-waist circumference (TyG-WC), TyG-waist to height ratio (TyG-WHtR), and TyG-body mass index (TyG-BMI) [13]. The diagnostic accuracy of these indices in metabolic syndrome has been shown [13]. The relationship of insulin resistance indices with the metabolic syndrome components, especially hypertension and prehypertension state, was investigated [9, 14–16]. In a study by Zeng et al., TyG, TyG‐BMI, TyG‐WC, and TyG‐WHtR presented positive correlations with systolic and diastolic blood pressure in individuals with proper body mass index [16]. In the research by Bala et al., TyG, TyG-BMI, and TyG-WC were independently associated with the presence of hypertension [9]. Moreover, Zheng and Mao identified TyG as the predictor of incident hypertension in a follow-up study [17].

A simple insulin resistance indicator TG/HDLc (triglicerides to high-density lipoprotein cholesterol), which is calculated as the ratio between triglycerides and high-density cholesterol concentrations, has also been proposed, and it was shown to be a marker for cardiometabolic and type 2 diabetes risk [18, 19]. It was also demonstrated that TG/HDLc is associated with hypertension [9].

Metabolic score for IR (METS-IR) is a novel score to assess insulin resistance defined as Ln((2 × fasting glucose) + fasting triglycerides) × body mass index)/(Ln(high-density lipoprotein cholesterol)) [20]. A significant correlation between METS-IR and intravisceral, intrahepatic (*ρ* = 0.636, *P* < 0.001) and intrapancreatic fat was presented [20]. METS-IR also correlated with fasting insulin levels [20]. The ability of METS-IR to predict type 2 diabetes in a 2-year follow-up study was evaluated, and it was presented that the group in the highest quartile of METS-IR had the highest risk to develop diabetes [20]. METS-IR was shown to correlate positively with blood pressure values and is strongly associated with hypertension in normal weight individuals [14]. These findings suggest that METS-IR may also be associated with metabolic syndrome. To date, the usefulness of METS-IR in the diagnosis of metabolic syndrome has not been studied.

Neck circumference (NC) is one of the anthropometric indicators of obesity, but also cardiovascular risk and metabolic syndrome [21, 22]. It seems that since the metabolic syndrome consists of excess body weight, insulin resistance, and hypertension, the combination of NC and TyG may appear more accurate than either of these indicators alone.

The study aimed to compare the usefulness of indirect insulin resistance indicators in detecting metabolic syndrome in non-diabetic patients, including the derivatives of TyG, and TG/HDLc. We also investigated the usefulness of METS-IR in the diagnosis of metabolic syndrome, which was for the first time to our knowledge. Then, we proposed novel indicators TyG-NC (TyG-neck circumference) and TyG-NHtR (Tyg-neck circumference to height ratio) and tested their usefulness in metabolic syndrome detection before the onset of diabetes. These new indicators are, to our knowledge, our concept and appear in medical literature for the first time.

## Materials and methods

The cross-sectional study included non-diabetic participants aged 18 and over. Individuals with previously diagnosed diabetes and fasting plasma glucose above 125 mg/dL were excluded from the study to analyze the group of patients before the onset of diabetes. Patients taking lipid-lowering drugs and individuals with severe hypertriglyceridemia (plasma triglycerides > 500 mg/dL) were also excluded from the study. The study was approved by the Ethical Committee of the Poznan University of Medical Sciences (approval number 359/15).

Clinical data of each patient was collected, and laboratory tests were performed.

### Laboratory tests

The fasting serum concentrations of glucose (FPG), triglycerides (TG), and high-density lipoprotein cholesterol (HDLc) were assessed.

### Anthropometric measurements and calculation of anthropometric indexes

The anthropometric measurements of each participant were carried out in the morning, fasting (at least 12 h after last meal):

- measurement of body height in an upright standing position without shoes with an accuracy of 0.5 cm,
- measurement of waist circumference (WC) at the approximate midpoint between the lower margin of the last palpable rib and the top of the iliac crest, using an unstrechable tape(in units of cm),
- measurement of neck circumference (NC) in the midway of the neck, between midcervical spine and midanterior neck, to within 0.5 cm, with plastic unstrechable tape
- measurement of body mass without shoes in underwear assessed using the certified electronic weighing scale with the accuracy of 0.1 kg (Radwag, Poland).
- body mass index (BMI), defined as the body mass divided by the square of the body height (expressed in units of kg/m^2^),
- waist to height ratio (WHtR), defined as the waist circumference divided by the body height
- neck to height ratio (NHtR) defined as the neck circumference divided by the body height

### Blood pressure measurements

The arterial blood pressure measurements were performed using Digital electronic tensiometer (Omron Corporation™, Kyoto, Japan), following the European Society of Hypertension and European Society of Cardiology (ESH/ESC) recommendations from 2013 [23]. Measurement was carried out twice, and if the values were significantly different, the measure was averaged out. Both systolic and diastolic blood pressure values were measured with an accuracy of 2 mmHg.

### Insulin resistance indexes

Insulin resistance indicators were calculated according to the following formulas:

TyG = Ln [fasting TG (mg/dL) × FPG (mg/dL)/2], [14]

TyG-BMI = TyG × BMI, [9]

TyG-WC = TyG × WC, [9]

TyG-WHtR = TyG × WHtR, [13]

TyG-NC = TyG × NC,

TyG-NHtR = TyG × NHtR,

TG/HDLc = fasting TG (mg/dL)/fasting HDL cholesterol (mg/dL), [14]

METS-IR = Ln [(2 × FPG (mg/dL) + fasting TG (mg/dL)] × BMI (kg/m^2^))/(Ln[HDLc (mg/dL]). [20]

### Metabolic syndrome criteria

Metabolic syndrome criteria were defined as the presence of any 3 of 5 risk factors established by the International Diabetes Federation Task Force on Epidemiology and Prevention in 2009 for the Caucasians [24]:

- triglycerides: ≥ 150 mg/dL or specific treatment for this lipid abnormality
- HDL cholesterol: < 40 mg/dL in males, < 50 mg/dL in females, or specific treatment for this lipid abnormality
- blood pressure (BP): systolic BP > 130 or diastolic BP > 85 mm Hg, or treatment of previously diagnosed hypertension
- fasting plasma glucose (FPG): ≥ 100 mg/dL or previously diagnosed type 2 diabetes (the individuals diagnosed with diabetes were excluded in this study)
- waist circumference ≥ 80 cm in females and ≥ 94 cm in males [24]

### Statistical analysis

Statistical analysis was performed with the Statistica v13 and Microsoft Excel Analyze it software. The predictive accuracy of insulin resistance indexes was assessed using the receiver operating characteristic curves (ROC curves). For each of the insulin resistance indexes, the calculations of the area under the ROC curve (AUC-ROC) were made. If the AUC was significantly greater than 0.5, the calculations of sensitivity and specificity were carried out. The optimal thresholds for each index were selected, choosing the highest Youden’s index value. First, a non-grouping analysis was performed, then curves were plotted, and calculations were made for normal BMI group (BMI between 18.5 and 24.99 kg/m^2^), overweight (BMI between 25 and 29.99 kg/m^2^) and obese group (BMI ≥ 30 kg/m^2^), respectively. The curves were plotted also separately for males and females to establish separate thresholds.

The minimal sample size was estimated using MedCalc software for the area under the ROC curve test. Based on previous reports, we assumed the predicted AUC as 0.75, type I error as 0.05, type II error as 0.20 [12, 13]. We estimated the ratio of negative to positive sample sizes as 0.6. The required sample size was estimated as 40, which we considered the minimum number of patients in each analyzed subgroup by gender and BMI.

## Results

The study included 665 individuals. Females constituted 70.4% of the group. Obesity was diagnosed in 26.5% of the group. Impaired fasting plasma glucose was present in 19.4% of the study group. 31.4% of the examined group were previously diagnosed with hypertension. 35.2% met the criteria of the metabolic syndrome. The values of the anthropometric, laboratory, and insulin resistance indices are presented in the Table 1.

**Table 1:** Group characteristics

Feature	Mean ± SD	Median
Age (years)	53.9 ± 14.5	57.0
Body mass (kg)	75.4 ± 15.2	73.5
Height (cm)	165.8 ± 9.1	165.0
Waist circumference (cm)	92.9 ± 14.2	92.0
Neck circumference (cm)	36.2 ± 3.7	36.0
BMI (kg/m^2^)	27.4 ± 4.8	26.9
WHtR	0.56 ± 0.08	0.56
NHtR	0.22 ± 0.02	0.22
FPG (mg/dL)	90.4 ± 11.1	89.0
TC (mg/dL)	203.6 ± 42.0	201.0
HDLc (mg/dL)	65.9 ± 17.2	63.0
LDLc (mg/dL)	111.1 ± 39.7	106.0
TG (mg/dL)	142.2 ± 78.2	125.0
Systolic BP (mmHg)	133.6 ± 19.3	132.0
Diastolic BP(mmHg)	80.9 ± 11.1	80.0
TyG	8.6 ± 0.6	8.6
TyG-BMI	236.7 ± 48.4	231.6
TyG-WC	803.9 ± 146.8	802.3
TyG-WHtR	4.9 ± 0.9	4.8
TyG-NC	313.0 ± 42.3	309.5
TyG-NHtR	1.9 ± 0.2	1.9
METS-IR	38.2 ± 8.6	37.3
TG/HDLc	2.4 ± 1.7	1.96

**Fig. 1:**
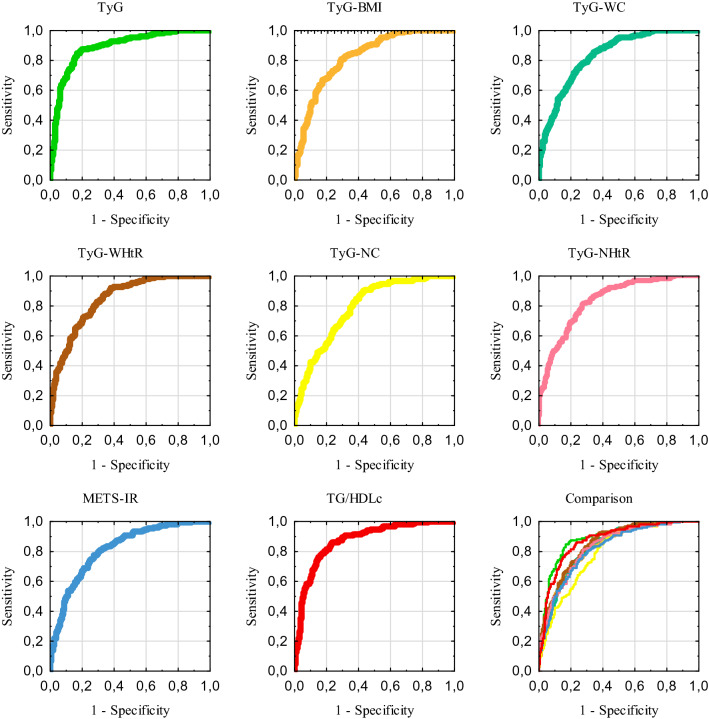
Receiver operating characteristic curves non-grouping analysis

The results of ROC-AUC analysis, 95% confidence interval, optimal thresholds, corresponding sensitivity, specificity, and Youden’s index values for each insulin resistance index for the whole study group are presented in the Table 2. The receiver operating curves for the tested indices and the comparison of them are presented in the Fig. 1. Testing of ROC-AUC showed that all analyzed insulin resistance indexes may discriminate the metabolic syndrome from healthy individuals. The analysis presented the highest area under the curve for TyG and TG/HDLc and the lowest area under the curve for TyG-NC.

**Table 2:** The receiver operating characteristic curve non-grouping analysis for insulin resistance indexes (*N* = 665)

Index	AUC	95% CI	*P* value	Threshold	Sensitivity	Specificity	Youden’s index
TyG	0.888	0.862–0.915	< 0.0001	8.795	0.850	0.828	0.679
F	0.891	0.859–0.918	< 0.0001	8.795	0.845	0.837	0.682
M	0.882	0.829–0.924	< 0.0001	8.756	0.904	0.798	0.702
TyG-BMI	0.826	0.795–0.857	< 0.0001	235.228	0.808	0.710	0.518
F	0.825	0.787–0.858	< 0.0001	231.802	0.795	0.723	0.518
M	0.841	0.782–0.889	< 0.0001	250.751	0.726	0.823	0.549
TyG-WC	0.832	0.801–0.862	< 0.0001	794.327	0.855	0.657	0.511
F	0.846	0.810–0.877	< 0.0001	782.362	0.839	0.710	0.549
M	0.852	0.795–0.899	< 0.0001	847.526	0.932	0.629	0.561
TyG-WHtR	0.847	0.818–0.876	< 0.0001	4.768	0.885	0.657	0.541
F	0.845	0.809–0.877	< 0.0001	4.664	0.907	0.652	0.558
M	0.857	0.800–0.903	< 0.0001	5.176	0.740	0.815	0.554
TyG-NC	0.791	0.757–0.825	< 0.0001	297.495	0.902	0.568	0.470
F	0.848	0.812–0.879	< 0.0001	297.199	0.870	0.736	0.606
M	0.826	0.766–0.876	< 0.0001	346.636	0.904	0.686	0.590
TyG-NHtR	0.831	0.800–0.862	< 0.0001	1.901	0.816	0.717	0.533
F	0.843	0.807–0.875	< 0.0001	1.874	0.814	0.746	0.560
M	0.842	0.783–0.890	< 0.0001	1.970	0.877	0.661	0.538
METS-IR	0.817	0.785–0.850	< 0.0001	38.447	0.774	0.726	0.500
F	0.822	0.784–0.856	< 0.0001	36.889	0.801	0.717	0.518
M	0.824	0.763–0.874	< 0.0001	40.535	0.795	0.718	0.512
TG/HDLc	0.874	0.847–0.902	< 0.0001	2.104	0.855	0.768	0.623
F	0.881	0.848–0.909	< 0.0001	1.871	0.882	0.752	0.634
M	0.878	0.824–0.920	< 0.0001	3.286	0.726	0.903	0.629

*AUC* area under the curve, *CI* confidence interval, *F* females (*N* = 468), *M* males (*N* = 197)

**Fig. 2:**
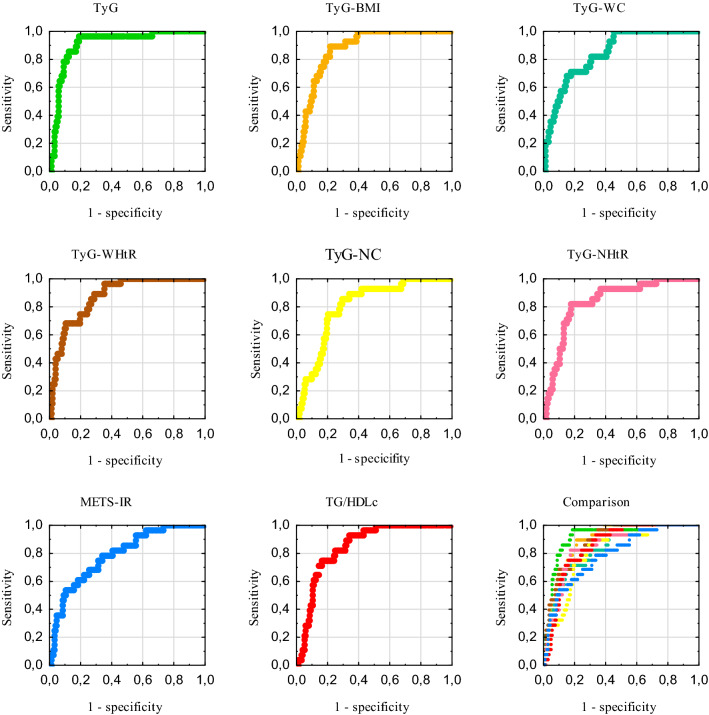
Receiver operating characteristic curves in the group with proper body mass index

The results of the ROC-AUC, 95% confidence intervals, optimal thresholds, corresponding sensitivity, specificity, and Youden’s index values for insulin resistance indexes in the group with proper body mass index are presented in the Table 3. All indexes achieved significant diagnostic accuracy, with the highest AUC for TyG and the lowest for METS-IR. Only the AUC for METS-IR in males did not reach statistical significance. The receiver operating curves for this group are shown in the Fig. 2.

**Table 3:** The receiver operating characteristic curve analysis for insulin resistance indexes in the group with proper body mass index (*N* = 224)

Index	AUC	95% CI	*P* value	Threshold	Sensitivity	Specificity	Youden’s index
TyG	0.909	0.856–0.962	< 0.0001	8.790	0.964	0.811	0.776
F	0.923	0.874–0.957	< 0.0001	8.790	0.958	0.823	0.781
M	0.868	0.728–0.953	< 0.0001	8.888	1.000	0.790	0.790
TyG-BMI	0.879	0.829–0.929	< 0.0001	198.424	0.893	0.786	0.679
F	0.891	0.836–0.932	< 0.0001	198.424	0.875	0.798	0.673
M	0.836	0.689–0.932	< 0.0001	198.183	1.000	0.737	0.737
TyG-WC	0.842	0.777–0.908	< 0.0001	732.232	0.714	0.827	0.541
F	0.851	0.791–0.900	< 0.0001	664.579	1.000	0.614	0.614
M	0.941	0.822–0.990	< 0.0001	825.116	1.000	0.921	0.921
TyG-WHtR	0.874	0.819–0.930	< 0.0001	4.136	0.964	0.648	0.612
F	0.867	0.808–0.912	< 0.0001	4.136	0.958	0.639	0.598
M	0.914	0.786–0.978	< 0.0001	4.329	1.000	0.816	0.816
TyG-NC	0.805	0.732–0.879	< 0.0001	293.288	0.893	0.704	0.561
F	0.859	0.800–0.906	< 0.0001	289.188	0.875	0.779	0.654
M	0.849	0.704–0.940	< 0.0001	338.735	1.000	0.816	0.816
TyG-NHtR	0.840	0.768–0.912	< 0.0001	1.837	0.821	0.821	0.643
F	0.852	0.791–0.900	< 0.0001	1.837	0.792	0.854	0.646
M	0.836	0.689–0.932	< 0.0001	1.844	1.000	0.711	0.711
METS-IR	0.789	0.704–0.875	< 0.0001	30.601	0.786	0.663	0.449
F	0.813	0.748–0.867	< 0.0001	30.601	0.792	0.715	0.507
M	0.697	0.536–0.829	0.0874	32.676	0.750	0.658	0.408
TG/HDLc	0.849	0.790–0.908	< 0.0001	1.547	0.929	0.658	0.587
F	0.861	0.802–0.908	< 0.0001	1.547	0.917	0.684	0.600
M	0.829	0.681–0.927	< 0.0001	2.131	1.000	0.737	0.737

*AUC* area under the curve, *CI* confidence interval, *F* females (*N* = 182), *M* males (*N* = 42).

**Fig. 3:**
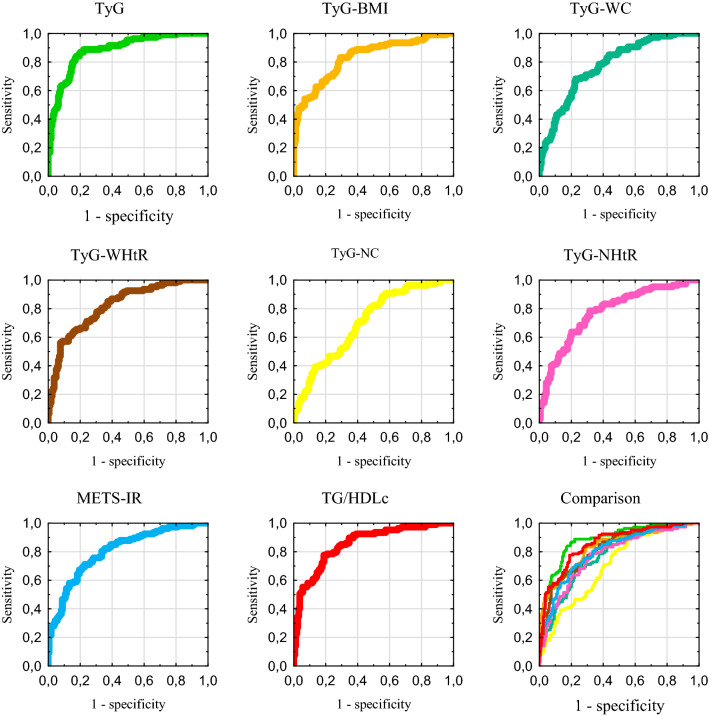
Receiver operating characteristic curves in the overweight group

The results of the ROC curve analysis for the overweight group are shown in the Table 4. As in the previous group, all indicators showed a significant AUC. The highest AUC was achieved by TyG and TG/HDLc, and the lowest by TyG-NC. The receiver operating curves for this group are demonstrated in the Fig. 3.

**Table 4:** Results of the receiver operating characteristic curve analysis for insulin resistance indexes in the overweight group (*N* = 265)

Index	AUC	95% CI	*P* value	Threshold	Sensitivity	Specificity	Youden’s index
TyG	0.884	0.840–0.920	< 0.0001	8.741	0.869	0.798	0.667
F	0.880	0.819–0.926	< 0.0001	8.688	0.864	0.794	0.657
M	0.899	0.826–0.949	< 0.0001	8.754	0.927	0.803	0.730
TyG-BMI	0.831	0.781–0.874	< 0.0001	235.228	0.832	0.709	0.541
F	0.826	0.757–0.881	< 0.0001	236.525	0.773	0.772	0.545
M	0.852	0.770–0.913	< 0.0001	235.136	0.902	0.652	0.554
TyG-WC	0.775	0.720–0.824	< 0.0001	836.303	0.682	0.772	0.454
F	0.819	0.750–0.875	< 0.0001	790.917	0.773	0.750	0.523
M	0.830	0.745–0.895	< 0.0001	847.526	0.927	0.621	0.548
TyG-WHtR	0.821	0.769–0.865	< 0.0001	4.798	0.851	0.627	0.477
F	0.816	0.746–0.873	< 0.0001	4.798	0.818	0.685	0.503
M	0.839	0.755–0.903	< 0.0001	4.977	0.805	0.727	0.532
TyG-NC	0.705	0.646–0.759	< 0.0001	296.451	0.888	0.443	0.331
F	0.802	0.731–0.861	< 0.0001	296.451	0.833	0.717	0.550
M	0.803	0.715–0.873	< 0.0001	346.427	0.878	0.727	0.605
TyG-NHtR	0.775	0.720–0.824	< 0.0001	1.909	0.785	0.684	0.469
F	0.789	0.717–0.850	< 0.0001	1.909	0.682	0.815	0.497
M	0.823	0.737–0.890	< 0.0001	1.956	0.854	0.682	0.536
METS-IR	0.804	0.751–0.850	< 0.0001	38.922	0.710	0.772	0.482
F	0.829	0.761–0.884	< 0.0001	37.229	0.803	0.750	0.553
M	0.813	0.726–0.882	< 0.0001	40.535	0.707	0.803	0.510
TG/HDLc	0.855	0.807–0.895	< 0.0001	2.432	0.776	0.810	0.586
F	0.861	0.797–0.910	< 0.0001	1.871	0.879	0.728	0.607
M	0.880	0.803–0.935	< 0.0001	3.286	0.732	0.909	0.641

*AUC* area under the curve, *CI* confidence interval, *F* females (*N* = 158), *M* males (*N* = 107).

**Fig. 4:**
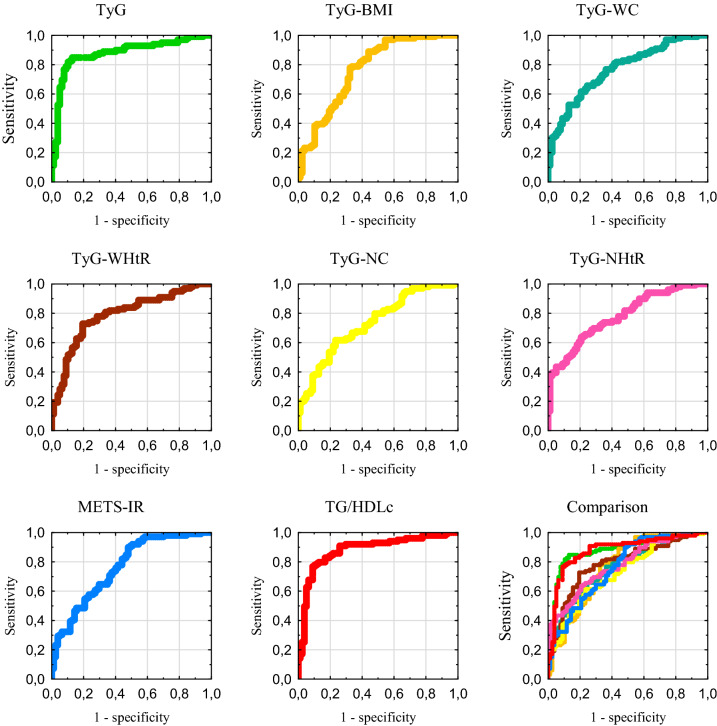
Receiver operating characteristic curves in the obese group

The results of the ROC curve testing in the group with obesity are presented in the Table 5. All indexes achieved significant diagnostic accuracy, with the highest AUC for TG/HDLc and TyG, and the lowest for TyG-NC. The receiver operating curves for this group are showed in the Fig. 4.

**Table 5:** Results of the receiver operating characteristic curve analysis for insulin resistance indexes in the obese group (*N* = 176)

Index	AUC	95% CI	*P* value	Threshold	Sensitivity	Specificity	Youden’s index
TyG	0.877	0.819–0.922	< 0.0001	8.765	0.849	0.870	0.719
F	0.870	0.799–0.923	< 0.0001	8.813	0.817	0.895	0.712
M	0.887	0.763–0.960	< 0.0001	8.719	0.857	0.900	0.757
TyG-BMI	0.798	0.698–0.828	< 0.0001	287.928	0.778	0.675	0.453
F	0.749	0.665–0.822	< 0.0001	275.782	0.887	0.544	0.431
M	0.814	0.676–0.912	< 0.0001	287.116	0.786	0.750	0.536
TyG-WC	0.767	0.697–0.827	< 0.0001	951.726	0.657	0.753	0.410
F	0.779	0.697–0.847	< 0.0001	920.556	0.704	0.755	0.459
M	0.825	0.688–0.919	< 0.0001	987.361	0.857	0.800	0.657
TyG-WHtR	0.789	0.721–0.846	< 0.0001	5.745	0.727	0.792	0.520
F	0.769	0.686–0.839	< 0.0001	5.668	0.747	0.754	0.501
M	0.850	0.717–0.937	< 0.0001	5.745	0.821	0.800	0.621
TyG-NC	0.732	0.660–0.796	< 0.0001	339.246	0.616	0.766	0.382
F	0.786	0.704–0.853	< 0.0001	333.076	0.549	0.877	0.426
M	0.775	0.631–0.883	< 0.0001	363.852	0.786	0.650	0.436
TyG-NHtR	0.779	0.710–0.838	< 0.0001	2.081	0.636	0.792	0.429
F	0.785	0.703–0.852	< 0.0001	2.045	0.648	0.772	0.420
M	0.816	0.678–0.913	< 0.0001	2.206	0.607	0.950	0.557
METS-IR	0.762	0.692–0.823	< 0.0001	44.425	0.899	0.520	0.419
F	0.747	0.662–0.819	< 0.0001	43.215	0.958	0.491	0.449
M	0.811	0.672–0.909	< 0.0001	47.961	0.750	0.800	0.550
TG/HDLc	0.880	0.823–0.924	< 0.0001	2.720	0.768	0.909	0.677
F	0.877	0.807–0.929	< 0.0001	2.489	0.775	0.930	0.705
M	0.902	0.781–0.969	< 0.0001	3.152	0.821	0.950	0.771

*AUC*A indicate area under the curve, *CI* confidence interval, *F* females (*N* = 128), *M* males (*N* = 48)

## Discussion

Our study confirms previous reports that insulin resistance’s indirect indices may be of diagnostic value in diagnosing metabolic syndrome [12, 13, 25]. Interestingly, it was demonstrated that TyG has a better capacity in predicting metabolic syndrome than HOMA-IR, which emphasizes the role of indirect indices of insulin resistance in daily practice [10, 12]. Several studies have compared the usefulness of insulin resistance indicators [13, 25, 26]. Yu et al. compared TyG, METS-IR, and TG/HDLc in diagnosing metabolic syndrome, identifying TyG as the one with the highest diagnostic accuracy, followed by TG/HDLc and METS-IR, which is consistent with our results [26]. A study comparing IR indices in metabolic syndrome by Raimi et al. showed the largest AUC for metabolic syndrome detection for TyG-WHtR, followed by TyG-WC, TyG-BMI, and eventually, TyG index, which is inconsistent with our study [13]. However, the discrepancy in the markers’ usefulness across countries has already been suggested and it is possible that ethnic differentiation can explain these differences [13]. A study by Lee et al. suggests TyG as an indicator of metabolically obese but normal weight, which is consistent with our observations that TyG is the best indicator for recognizing the metabolic syndrome features in a group of patients with normal body weight [5]. In the study by Lim et al., TyG-BMI was found to predict insulin resistance better than TyG, TyG-WC, and TyG-WHtR [25]. In this research, TyG and obesity indices’ combinations showed better insulin resistance prediction performance than TyG alone [25]. However, our study shows that in the diagnosis of metabolic syndrome, TyG has a better diagnostic value than its products with anthropometric indices. The studied group's characteristics may also be of some importance, as the mean BMI in the study mentioned above was lower than in ours, amounting 23.8 ± 3.1, which may partly explain the differences [25].

Neck circumference was suggested as a new promising indicator of metabolic syndrome and cardiovascular risk [21, 22, 27]. ROC curve analysis performed by Laohabut et al. showed that neck circumference may be a useful tool for metabolic syndrome prediction [22]. Yang et al. presented that larger neck circumference is associated with an increased risk of coronary heart disease [27]. Moreover, the neck circumference and waist circumference ability to predict cardiovascular risk and identify the presence of metabolic syndrome seems similar [28, 29]. Since NC and TyG appear to be good indicators of insulin resistance and metabolic syndrome, TyG-NC and TyG-NHtR should potentially be a good representation of the metabolic syndrome features. As expected, both TyG-NC and TyG-NHtR reached the areas under the curve, which makes them useful diagnostic tests. TyG-NHtR seems to have a higher diagnostic value than TyG-NC, however, the application of these two indicators in practice requires further observations.

TG/HDLc, despite its simplicity, seems to be just as useful, if not better, than some of the proposed indicators based on combined biochemical and anthropometric measurements. In our study, TG/HDLc achieved the second-largest ROC-AUC after TyG with high sensitivity and specificity in the whole study group, and the highest diagnostic accuracy in the group of obese individuals.

The study also has some limitations that need to be identified. First, the study was conducted on the Caucasian population, so one should carefully conclude on the usefulness of the investigated indexes in other populations. Moreover, to create a homogenous group of patients and to identify indicators useful in the early diagnosis of metabolic syndrome, patients taking medications for hyperlipidemia, impaired glucose tolerance and diabetes were excluded from the analysis. Therefore, the proposed indicators should be considered early markers of metabolic syndrome that are useful in patients prior to treatment. Separate studies are required to identify the indices useful in the diagnosis of metabolic syndrome in groups of patients during the treatment of impaired glucose tolerance, diabetes, or hyperlipidemia. Furthermore, the cut-off points presented may have been affected by unequal gender representation, and gender-specific thresholds should be considered more reliable. Finally, the reported cut-off points should be considered tentative due to the study group's insufficient size to consider the results as reference values for the entire population.

## Conclusions

Indirect indices of insulin resistance, such as TG/HDLc, METS-IR, TyG, and its derivatives, including newly proposed TyG-NC and TyG-NHtR, show an essential diagnostic value in the diagnosis of the metabolic syndrome. TyG and TG/HDLc seem to be the most useful in the Caucasian population, but further research is needed to clarify the use of individual indicators. TG/HDLc deserves additional emphasis, as calculated from just two biochemical parameters is not inferior to other indicators' diagnostic value, and its use in everyday medical practice should be considered.

## Data Availability

The datasets generated during and/or analyzed during the current study are available from the corresponding author on reasonable request.
